# Therapeutic Exercise Intervention Using Vibration Platforms for Glycemic Control in Type 2 Diabetes: A Pilot Study

**DOI:** 10.3390/jcm12206518

**Published:** 2023-10-14

**Authors:** Juan Fabregat-Fernández, Vicente Rodríguez-Pérez, Rocío Llamas-Ramos, Ana Felicitas López-Rodríguez, Jesús Seco-Calvo, Inés Llamas-Ramos

**Affiliations:** 1 Department of Nursing, University of Extremadura, 10600 Plasencia, Spain; juanfabregat@unex.es; 2 Faculty of Nursing and Physiotherapy, Universidad de Salamanca, 37007 Salamanca, Spain; inesllamas@usal.es; 3 Instituto de Investigación Biomédica de Salamanca (IBSAL), 37007 Salamanca, Spain; 4 Department of Nursing and Physiotherapy, Faculty of Nursing and Physiotherapy, Universidad de León, 24401 León, Spain; ana.lopez.rodriguez@unileon.es; 5 Institute of Biomedicine (BIOMED), University of Leon, 24071 León, Spain; jesus.seco@unileon.es; 6 Physiology Department, University of the Basque Country, 48940 Leioa, Spain; 7 Primary Care Research Unit of Salamanca (APISAL), 37005 Salamanca, Spain; 8 University Hospital of Salamanca, 37007 Salamanca, Spain; 9 Health Service of Castilla y Leon (SACyL), 47007 Valladolid, Spain

**Keywords:** diabetes mellitus type 2, nursing, physical exercise, whole-body vibration

## Abstract

Diabetes generates a great impact on society, as well as a concern for health professionals due to its high and increasing prevalence; there are several studies that demonstrate the effectiveness of vibration platforms and their benefits at a physiological level. The aim of this study will be to analyze the decrease in glycosylated hemoglobin and glycemia levels after the use of whole-body vibration platforms and the possible inclusion of this therapeutic option within the usual treatments. This is a double-blind, randomized controlled trial with parallel group design in a 1:1 ratio. The sample will be composed of people diagnosed with type 2 diabetes mellitus in in the Plasencia area (Cáceres, Extremadura). Participants will be randomly assigned to the intervention or control group using a randomization list and will follow the inclusion criteria: type 2 diabetics between 50 and 60 years of age and not taking diabetes medication. All participants will undergo a determination of glycosylated hemoglobin, blood pressure, lipid profile, weight and height, and different functional tests such as Time Up and Go, 10 Meters Walk Test, and 5 Sit To Stand. The experimental group will perform a whole-body vibration intervention on an oscillating platform for 12 weeks with a weekly frequency of three nonconsecutive days and a duration of 12 min. The exercises will consist of 60 s of work and 60 s with rest. The control group will carry out their normal life insisting on the importance of glycemic controls before and after their daily physical exercise. This study has been registered at clinical.trial.org, ID: NCT 05968222. Whole-body vibration platforms have demonstrated their effectiveness in different pathologies such as stroke, fibromyalgia, sclerosis multiple, or Parkinson’s. For that reason, an improvement in glycemic and lipid values and body composition are expected in people with diabetes after a whole-body vibration intervention for 12 weeks’ duration. In addition, whole-body vibration platforms could be postulated as an alternative to usual treatments.

## 1. Introduction

The human body is subjected to constant vibrations during daily activities that generate a series of negative physiological responses in the organism [1]. The use of vehicles, sports performance, or professional activity with machinery such as hammers can cause injuries. The common characteristics of these vibrations are high intensity, high frequency, and long exposure times. However, the exposure of the human body to low-intensity vibrations and short exposure times has been shown as a training method to be proved to be a tool for improvement of strength and co-ordination [2]. 

In addition, vibration therapy (VT) interventions can be an attractive alternative for people with contraindications to physical activity or manifesting kinesiophobia [3].

There are basically three types of VT: first, the vibration is applied directly to the muscle or the tendon of a muscle by a vibration device or by vibrating cables [4,5] (direct vibration); second, the vibration enters the human body through the hands when gripping a vibrating dumbbell or bar (indirect vibration) (both types of segmental vibration) [5]; and, third, the vibration enters when standing on a vibration platform, so-called whole-body vibration (WBV) [6]

WBV can be applied in treatment using stimuli with specific parameters, such as frequency, amplitude, time, and propagation method [7]. The effects of WVB are based on the fact that vibrations activate muscle spindles, causing their contraction: tonic vibration reflex (TVR) [8]

There are numerous research studies in which WBV has demonstrated efficacy in both healthy populations and in those with health issues. Marín-Cascales et al., 2018 showed that WBV is an effective method in postmenopausal women with osteoporosis (OP), improving bone mineral density (measured in lumbar spine and femoral neck) [9].

It has been proved that vibration therapy applied regularly has a beneficial impact on lowering the level of total cholesterol and LDL cholesterol in people with hypercholesterolemia and in women aged 65 years or more [3].

Therefore, perhaps the use of vibration therapy can be very useful in the pathologies of the elderly, where the passage of time causes functional deterioration of organs, systems and tissues, balance disorders, falls, joint problems, and metabolic disorders (such as diabetes mellitus (DM) and obesity).

DM is one of the most prevalent pathologies. Specifically, worldwide, 536.6 million people suffer from diabetes; in Spain, the incidence of type 2 DM is estimated at one in seven people, which is equivalent to 13.8% of the population (around 5.1 million adults) [10].

DM is characterized by persistently elevated blood glucose levels as a result of impaired insulin secretion, insulin action, or both. According to the American Diabetes Association (ADA) [11], diabetes can be classified into:

Diabetes mellitus type 1 (DM 1): due to autoimmune destruction of β-cells, usually leading to absolute insulin deficiency. Diabetes mellitus type 2 (DM 2): caused by a progressive loss of insulin secretion from β-cells during the course of the disease. Gestational diabetes mellitus (GDM): diabetes diagnosed in the second or third trimester of pregnancy that was not clearly manifest diabetes before gestation. Specific types of diabetes due to other causes, e.g., monogenic diabetes syndromes (such as neonatal diabetes and maturity-onset diabetes of the young), diseases of the exocrine pancreas (such as cystic fibrosis), and drugs or chemicals. Finally, induced diabetes, such as with glucocorticoid use, in the treatment of HIV/AIDS or after organ transplantation.

Diabetes has a great impact on society, and health professionals are concerned about its high and ever-increasing prevalence, not to mention the high cost to the health care system; raising awareness among these people is the key to combating this problem [11].

This fact has led to an increasing number of studies demonstrating vibration platforms’ effectiveness and their benefits at a physiological level, since it has been proven that they improve glycosylated hemoglobin, plasma glycemia, flexibility, contribute to the reduction or elimination of pain, improve blood circulation, increase bone mineral density (BMD), among others [9,12].

Therefore, the following clinical trial is proposed to test the effectiveness of whole-body vibration platforms in type 2 diabetic patients as well as the choice of this physical activity treatment as a possible alternative or complement to the usual treatment.

## 2. Materials and Methods

The hypothesis of this study will be that the use of whole-body vibration has an effect on type 2 diabetes mellitus by decreasing the levels of glycosylated hemoglobin and blood glucose.

The objective of the study will be to analyze the decrease in glycosylated hemoglobin and blood glucose levels after the use of whole-body vibration platforms at baseline and at 12 weeks in people diagnosed with type 2 diabetes mellitus.

### 2.1. Study Design and Setup

The study is a double-blind, randomized controlled trial with parallel group design in a 1:1 ratio.

### 2.2. Participants

The study will be carried out with diabetic patients from the Plasencia area. Participants who wish to participate in the present study must meet the following inclusion criteria: healthy type 2 diabetic patients between the ages of 50 and 60 years who are not taking medication for diabetes. Exclusion criteria will be established as having some underlying pathology (such as diabetic retinopathy or diabetic nephropathy), mobility disability and/or comorbidities incompatible with physical exercise and the use of vibration platforms, previous hip or knee surgeries, cognitive impairment, and neurological pathology.

**Figure 1:**
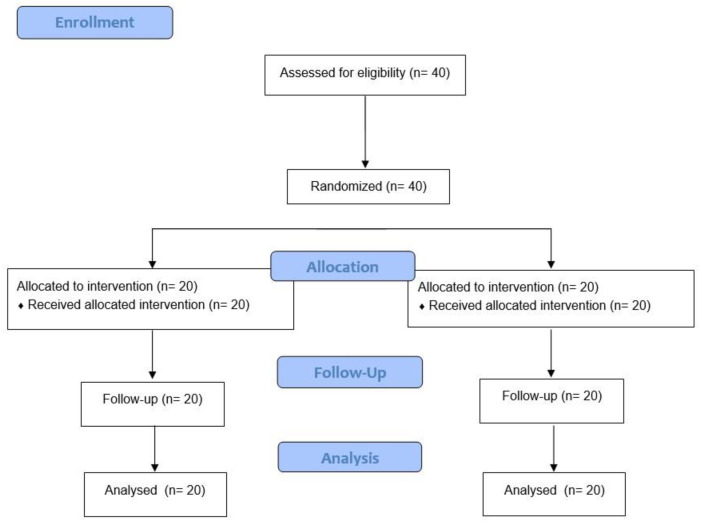
Flow diagram.

Given the lack of studies that allow us to calculate the sample size for the sample to be representative, a pilot study will be carried out to obtain these data. A total of 40 subjects (20 men and 20 women) will be evaluated, divided into a control group (n = 20) and an experimental group (n = 20) (Figure 1).

### 2.3. Variables

Anthropometric measurements such as weight, height, and waist–hip ratio will be taken. In addition, functional tests such as Time Up and Go (TUG), 10 Meters Walk Test (10MWT), and 5 Sit To Stand (STS) will be performed. Finally, capillary blood samples will be taken to measure HbA1c and plasma glucose and lipid profile. In addition, blood pressure, weight, and height values will be collectd.

HbA1c is a blood test to determine the average level of glucose or sugar in the blood during the last two or three months [11]. According to the ADA for the diagnosis of diabetes, HbA1c should be at values greater than or equal to 6.5% (≥48 mmol/mol); however, normal HbA1c values should be below 5.7%. The Affinity A1c Analycer^®^ (Debramedix Slne laboratory, Spain)will be used. The HbA1c analysis is a simple test, which consists of obtaining a few drops of capillary blood from a fingertip using a lancet and transferring them to the Affinity A1c Analycer^®^. HbA1c will be taken at baseline and at 12 weeks post procedure to identify variations after VBW.

Regarding capillary glucose, it is a capillary blood glucose measurement using a glucometer (FreeStyle InsuLinx glucometer^®^, Abbott laboratory, Spain) to determine glucose values and facilitate self-monitoring of DM. Obtaining a capillary blood glucose sample is necessary to verify that participants are in optimal conditions to perform the training session (blood glucose levels lower than 90 mg/dl or higher than 250 mg/dl prevent participants from performing the exercises) and also to verify the effect of whole-body vibration (WBV) on the behavior of blood glucose 48 h after the previous session [11].

The LUX^®^ meter (Debramedix Slne laboratory, Spain) will be used for the quantitative measurement of blood parameters such as total cholesterol, HDL cholesterol, and LDL cholesterol (calculated). As with the HbA1c, it is a simple test, which consists of obtaining a few drops of capillary blood from a finger of the hand using an auto-lancet Vitrex Sterilance (Debramedix Slne laboratory, Spain) and pouring them into the LUX^®^ meter. The lipid profile should be below 200 mg/dL for total cholesterol, 40 mg/dL or higher for HDL cholesterol, and less than 100 mg/dL for LDL cholesterol and should be taken at baseline and at 12 weeks after the start of the procedure to identify variations after VBW [13].

Blood pressure is the force of your blood pushing against the walls of your arteries and will be determined with an Aneroid Sphygmomanometer (Riester, Jungingen, Germany). The values of this parameter considered normal are those situated at 120 mmHg systolic pressure and 70 mmHg diastolic pressure. Blood pressure will be a parameter, together with capillary glycemia, necessary to indicate whether the participants are in optimal conditions to perform the training session [14].

Height will be determined with a “Seca” (seca Deutschland, Hamburg, Germany) portable detachable stadiometer and weight with a Marsden M550 portable scale (Flintec Scales laboratory SL, Spain).

Anthropometric measurements will always be taken in the same space and at the same time, an early hour in the morning, keeping an ambient temperature between 20 and 22 °C.

**Timed Up and Go 3 m (TUG 3 m)**

This test will be performed with the use of a specific application (Mon4tic for IOS system). The cell phone is placed on the sternum by means of a special belt or held in place by the subject. The subject, seated with his back resting on the back of the chair (without armrests and height 43 cm), at the acoustic command following a five-second countdown, stands up, walks the expected distance, turns around a cone, goes back, turns 360°, and sits down again. The test time stops when the subject returns to the starting position and is completely immobile [15].

**10 Meters Walking Test (10 m)**

On command, the subject covers the distance walking as fast as possible, without running [16].

**Five times Sit To Stand (5STS)**

The subject will be seated with his back supported on the chair (without armrests and at a height of 43 cm) and, on command, stand up and sit down five consecutive times, as fast as possible and without using the push of the hands on the quadriceps. The arms may be crossed over the chest and subjects are instructed to stand up completely and not to lie down on the chair between repetitions [17].

These three tests will be implementd once on the same day (beginning and after each session) to avoid clinical changes.

### 2.4. Vibration Platform

A horizontal oscillating platform (Galileo 2000©, CELDUAL, S.L., Valencia, Spain) with an amplitude of 2 mm and a constant frequency of 14 Hz will be used as a WBV instrument.

### 2.5. Procedure

Participants with type 2 diabetes will be recruited through talks, on alternate days, at the diabetic association of Plasencia (Cáceres). Those who wish to participate and meet the inclusion criteria will be contacted through a preliminary interview where they will be informed of the methodology, objectives, and process of the study and will be given an informed consent form that must be signed for their inclusion in the study. 

The screening will be performed by two physiotherapists and a research nurse at the University Center of Plasencia. The selected participants will undergo an interview where they will be asked about mobility disability and/or comorbidities incompatible with physical exercise in order to exclude those participants who do not meet the inclusion/exclusion criteria.

The study will be conducted from February 2024 to May 2024 by the aforementioned professionals. Participants will be randomly assigned to the intervention or control group, using a randomization list generated by a computer program. All participants who meet the above criteria will be offered entry into the study and will be given information and the schedule of interventions to participate in the study. Patients will continue with their usual lifestyle. After providing information about the study, they will be randomly included in one of the two groups (intervention or control).

Two of the researchers will be responsible for the correct control of the frequency and amplitude of the vibration platform during the entire intervention and for the correct performance of the exercises proposed for each training session, in addition to the functional tests TUG, 10MWT, and 5STS. A third researcher will be responsible for the determinations and parameters to be measured in the sample subjects (HbA1c and plasma glucose, lipid profile, weight, height, and blood pressure).

#### 2.5.1. Control Group

Participants in the control group will undergo a determination of glycosylated hemoglobin, blood pressure, lipid profile, weight and height, as well as functional tests such as TUG, 10MWT, and 5STS. Subsequently, they will be encouraged to continue their normal life, insisting on the importance of glycemic controls before and after their daily physical exercise, in addition to continuing with the dietary measures recommended by their specialist. After 12 weeks, glycosylated hemoglobin, blood pressure, lipid profile, weight, height, and the TUG, 10MMT, and 5STS tests will be redetermined.

#### 2.5.2. Experimental Group

Participants in the intervention group will be subjected to the following tests:

- At the beginning of the intervention, a determination of glycosylated hemoglobin, blood pressure, lipid profile, weight, height, and TUG, 10MWT, and 5STS, which will be repeated after 12 weeks of intervention.
- Before each training session, participants will undergo a basal blood glucose and blood pressure measurement, which will be repeated after the end of the session.
- Each training session on the vibrating platform is composed of 6 exercises, with a duration of 60 s, with 60 s pauses between exercises and a warm-up exercise:

**Warm-up exercise: classic squat**

Prior to the training session, the subjects will place their feet on the floor at shoulder height, with their arms stretched forward to gain stability; the knee will be reflected at 100° and the back will be kept straight, returning to the starting position.

1. 1. Alternating feet exercise.

With the back to the platform and the feet completely straight, the subjects will bend the knee while keeping the other leg straight. Then, they will alternate and do the same with the other leg for the duration of the exercise.

2. Up and down exercise.

Starting from the standing position on the platform, subjects will lower the right–left foot and then raise the right–left foot, repeating the exercise for 60 s.

3. Stride exercise

The subjects, starting from the initial standing position, will be separated at shoulder width and then place one foot on the platform, bending the knee at 90°, and the other on the floor. The exercise will be carried out with both legs alternately, holding the handlebars of the platform to maintain balance.

4. Heel Raise Exercise

The subjects, starting from the initial standing position, will be separated at shoulder width and raise their heels, keeping their knees slightly bent, their back straight, their eyes straight ahead, and holding the handlebars of the platform to maintain their balance.

5. Squat exercise

Starting from a standing position, shoulder-width apart, the subject will perform a 100° knee flexion, returning to the starting position.

6. Squat exercise with weight changes.

Starting from the squat position, the subject will perform a knee flexion–extension while shifting body weight in the mid-lateral axis (left–right) alternately.

### 2.6. Study Schedule

During the first week of February, detailed information on the procedure, informed consent, and the schedule of the study interventions will be provided. 

During the second week of February, preintervention measurements of HbA1c, lipid profile, blood pressure, height and weight, and TUG, 10MWT, and 5STS will be carried out so that they can be compared with the postintervention measurements.

During the following 12 weeks, the experimental group will carry out the intervention by means of the exercises in the WBV. These exercises will be performed three days a week, with a rest of at least 24 h between days of intervention.

**Figure 2:**
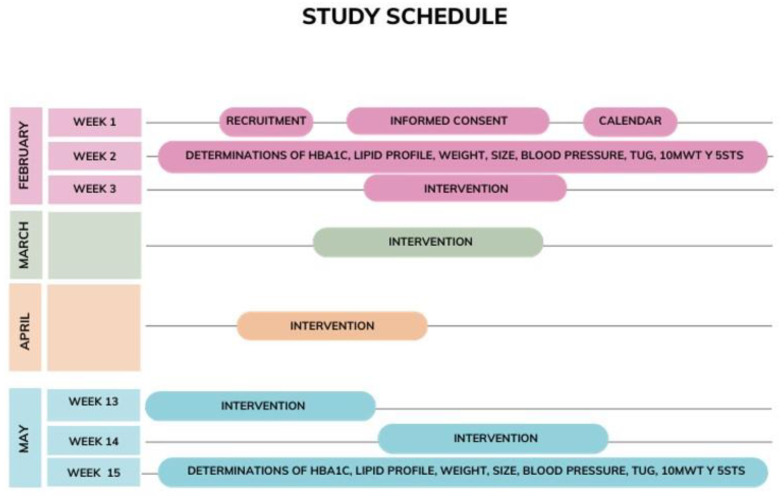
Study schedule.

In the third week of May, after the end of the intervention, the participants will again undergo HbA1c, lipid profile, weight, height, and TUG, 10MWT, and 5STS tests. The control group will also have their blood pressure measured (Figure 2).

### 2.7. Statistical Analysis

A descriptive analysis will be performed for the experimental and control groups. Similarity between groups will be calculated at baseline and differences between groups at 12 weeks will be examined. The variables HbA1c, lipid profile, weight, height, and blood pressure will be selected in bivariate analyses. Adjustment for confounding factors will be examined by linear regression. Questionnaire scores will be assessed and changes between baseline and 12 weeks between groups will be sought. Statistical analysis will be performed using IBM SPSS statistics version 28.0.

### 2.8. Ethical Considerations

The study will be implemented in accordance with the Declaration of Helsinki and the ethical guidelines for medical research in humans. This study has been approved by the Bioethics and Biosafety Committee of the University of Extremadura (Registration No. 101//2023) and it has been registered at clinical.trial.org with the ID: (NCT 05968222). No patient identifiable information will be stored. Only the investigators will have access to the final data set of the study. It is considered that harm to the patient will be unusual because the tests performed will be those performed in the usual medical examination. However, participants will be free to leave the study if they wish to do so. Discontinuation of participation in the study may be made if the participant wishes to withdraw consent to participate, if the physician determines that continuation of the intervention is not indicated, or if the participant is unable to follow the study protocol. The likelihood of any health hazard during the procedure is minimal. If any serious adverse events occur, they will be reported according to the standard procedure for reporting serious adverse events in clinical research.

## 3. Discussion

Aerobic physical exercise of moderate intensity is recognized as an effective nonpharmacological approach to reduce glycemia. The physiological modifications observed with WBV are analogous to those of any physical activity and, in addition to acute changes, chronic adaptations in the mechanical behavior of the muscles have been described. It is known that muscles react to vibration by contracting and stretching automatically. Vibration triggers a muscle contraction reflex, the TVR, which causes an increase in hormone secretion, among others [18]. That is why WBV could be a type of physical exercise to reduce glycemia and HbA1c, in addition to lipid profile, weight, height, functional balance, and neuropathic pain [12,19,20,21,22]. However, studies such as that of Behboudi et al. [23], after 12 weeks of whole-body vibration intervention, did not show a significant reduction in HbA1c. Another study by Liphardt et al. points in the same direction that bone quality in osteopenic postmenopausal women is not improved after 12 months of whole-body vibration training [24].

It is important to differentiate between vibratory devices that subject the body to vibration while the body is kept relaxed or resting on couches or beds (oscillatory-cyclic and oscillatory-cycloid vibration [3,12]) and WVB devices where the patient performs a voluntary exercise while adding the vibratory stimulus, affecting the whole body placed on top of a vibrating platform. It is a voluntary and conscious exercise to which the vibratory stimulus is added.

Since it is a physical exercise different from that practiced by the general population, it is new, and the sessions are of short duration, it could be estimated that the abandonment of the sample will only occur due to a very justified cause, which will reduce the negative results of the research.

HbA1C is one of the most determinant parameters in the diagnosis and long-term control of DM. According to the ADA [4], an HbA1c > 6.5% is considered a diagnostic criterion for DM, while values < 7.0% HbA1c determine good blood glucose control in the last four months.

This fact would behave as a protective factor in the development of certain cardiovascular diseases in people with DM 2, since it has been shown that the reduction in HbA1c in blood reduces the risk of developing heart disease by around 14% and, in addition, the risk of mortality [25,26].

Vibration platforms have been shown in different studies to be an effective tool for improving functional balance in healthy, physically active people [27,28].

Vibration platforms are shown to be effective in maintaining and improving balance in people with stroke [29], fibromyalgia [30,31], gait in Parkinson’s disease [32], and knee extension strength in multiple sclerosis [33]; however, according to their study, Slatkovska et al. [34] found WBV did not alter bone structure in postmenopausal women.

Although this protocol focuses on the use of WBV and its influence on glycosylated hemoglobin, this same intervention has also been shown to be effective in modifying other parameters, such as increasing blood flow and reduction in adiposity in patients with type 2 diabetes mellitus [22], as a complementary treatment in peripheral neuropathic pain due to diabetes (as a complimentary treatment in patients with diabetic peripheral neuropathic pain) [21]. On the other hand, Vry et al. [35] showed that the use of these devices has been shown to be a safe alternative in the rehabilitation of neuromuscular disorders.

Following the benefits provided in previous studies regarding the use of WBV, it is expected to improve glycemic, lipid, and body composition levels after the use of whole-body vibration for 12 weeks’ duration.

### Study Limitations

Among the possible limitations contemplated in the present study are the diversity of the different footwear of each patient, the nutritional habits of the participants due to not being able to follow an exhaustive control of the diet consumed, and the influence of climate on HbA1c. According to the study by Tseng et al. [36], it will be concluded that HbA1c has higher values depending on the season of the year; thus, during the winter, the values are higher than during warmer seasons. Our work will be performed during the winter and spring, so the different results may be influenced by these seasons.

## 4. Conclusions

The whole-body vibration intervention for type 2 diabetics will allow testing of its influence on glycosylated hemoglobin and capillary glycemia levels, lipid levels after the intervention, as well as on blood pressure, body mass index, and functional balance. In addition, the aim is to test the effectiveness of the whole-body vibration platform as a method of physical exercise, improving patient adherence and its use in health centers.

## Data Availability

All data will be available under reasonable request to the corresponding author.
